# NaHS Protects Cochlear Hair Cells from Gentamicin-Induced Ototoxicity by Inhibiting the Mitochondrial Apoptosis Pathway

**DOI:** 10.1371/journal.pone.0136051

**Published:** 2015-08-21

**Authors:** Yaodong Dong, Dongliang Liu, Yue Hu, Xiulan Ma

**Affiliations:** Department of Otology, Shengjing Hospital Affiliated to China Medical University, Shenyang 110004, Liaoning, China; Universitat Pompeu Fabra, SPAIN

## Abstract

Aminoglycoside antibiotics such as gentamicin could cause ototoxicity in mammalians, by inducing oxidative stress and apoptosis in sensory hair cells of the cochlea. Sodium hydrosulfide (NaHS) is reported to alleviate oxidative stress and apoptosis, but its role in protecting aminoglycoside-induced hearing loss is unclear. In this study, we investigated the anti-oxidant and anti-apoptosis effect of NaHS in *in vitro* cultured House Ear Institute-Organ of Corti 1 (HEI-OC1) cells and isolated mouse cochlea. Results from cultured HEI-OC1 cells and cochlea consistently indicated that NaHS exhibited protective effects from gentamicin-induced ototoxicity, evident by maintained cell viability, hair cell number and cochlear morphology, reduced reactive oxygen species production and mitochondrial depolarization, as well as apoptosis activation of the intrinsic pathway. Moreover, in the isolated cochlear culture, NaHS was also demonstrated to protect the explant from gentamicin-induced mechanotransduction loss. Our study using multiple *in vitro* models revealed for the first time, the potential of NaHS as a therapeutic agent in protecting against aminoglycoside-induced hearing loss.

## Introduction

In all mammals, hair cells are the sensory receptors of both the auditory and the vestibular system. Cochlear hair cells grow in an array of four rows in organ of Corti along the entire length of the cochlear coil. The outer three rows consist of outer hair cells (OHCs), and they function as the cochlea's mechanotransduction amplifier. The other row of cells close to the center of cochlea is called inner hair cells (IHCs), and provides the main neural output of the cochlea [1, 2]. Because hair cells are terminally differentiated and are incapable of regeneration, damages to them result in reduced sensitivity of hearing and in severe cases complete sensorineural hearing loss [3]. Sensorineural hearing loss is a prevalent worldwide health problem, and a significant proportion of hearing loss is caused by aminoglycoside-induced death of sensory hair cells [4]. Aminoglycosides, such as gentamicin, amikacin, kanamycin and neomycin, originate generally from Gram-negative bacteria and is able to inhibit protein synthesis [5]. However aminoglycosides were widely reported to cause ototoxicity [6], and induce intrinsic apoptosis of hair cells through oxidative stress in birds and zebrafish [7, 8].

Apoptosis occurs through two different signaling pathways: the intrinsic and extrinsic pathways [9]. The extrinsic pathway of apoptosis involves the tumor necrosis factor (TNF) receptor gene superfamily, which function as transmembrane death receptors [10]. These receptors can be activated by extrinsic ligands, such as FasL and TNF-α, and results in the activation caspase-8 and the ultimate destruction of the cell [11]. On the other hand, the intrinsic (mitochondrial death) pathway of apoptosis is regulated by the combined actions of the pro- and anti-apoptotic members of the Bcl-2 family proteins [12]. Among them, Bcl-2-like protein 4 (Bax) is a major pro-apoptotic member, whose activation results in the release of pro-death proteins from the intermembrane space of the mitochondria into the cytosol. On the contrary, the anti-apoptotic protein Bcl-2 inhibits apoptosis by preventing the activation of inner mitochondrial permeability transition pore and release of pro-apoptogenic mitochondrial contents including cytochrome *c* [12]. When released into the cytoplasm, cytochrome *c* recruits caspase-9 which in turn induces caspase-3 dependent apoptosis [13]. In most mammalian cells, the mitochondria produces reactive oxygen species (ROS), such as hydroxyl radicals, superoxide anions and hydrogen peroxide [14]. ROS, toxic byproducts of cellular metabolism from many cytoplasmic sources, can function as signaling molecules that regulate many physiological processes including the intrinsic apoptosis pathway [15]. Accumulation of ROS in the cells leads to cellular oxidative stress, and is considered one major initiator of aminoglycoside-induced ototoxicity [16].

Hydrogen sulfide (H_2_S) is commonly considered as a toxic gas and environmental pollutant with an offensive odor. However in recent years, its protective role as an oxygen scavenger to antagonize oxidative stress, inflammation and apoptosis has gained much attention. As an endogenous donor of H_2_S, NaHS was found to protect cardio-myoblasts against oxidative challenge through the inhibition of L-type calcium channels in a rat model [17]. NaHS also exhibits anti-inflammatory effect in post-ischemic murine small intestine [18, 19]. Of particular interest to our current study, NaHS was reported to prevent apoptosis in the mouse brain by attenuating caspase-3 activation [20]. However, no study has been performed on the effect of NaHS in aminoglycoside-induced hearing loss.

In this study, we aimed to investigate whether the reported anti-oxidant and anti-apoptosis effect of NaHS could be applied in the protection against gentamicin-induced ototoxicity. Using House Ear Institute-Organ of Corti 1 (HEI-OC1) cell line and mouse cochlear tissue explant as models, we studied the effect of NaHS in protecting them after gentamicin insult. We demonstrated that NaHS significantly reduced gentamicin ototoxicity in both HEI-OC1 cell and cochlear explant cultures, by inhibiting the intrinsic caspase-3 apoptotic pathway. Our study provided the first instance supporting the use of NaHS as a potential therapeutic agent to protect against aminoglycoside-induced hearing loss.

## Materials and Methods

### Cell line, animals and ethics statement

House Ear Institute-Organ of Corti 1 (HEI-OC1) cell line was derived from the cochlea of the Immorto-mouse and characterized by Kalinec *et al* [21]. Cells were maintained in high-glucose Dulbecco’s modified Eagle’s medium (DMEM; Gibco) supplemented with 10% fetal bovine serum (FBS; Gibco) at 33°C in a humidified incubator with 5% CO_2_.

The Atoh1/GFP transgenic mice carrying GFP under the control of Atoh1 enhancer [22] were purchased from Cyagen, Shanghai. All procedures and experiments involving animals in this study were performed in accordance with the National Institutes of Health Guide for Care and Use of Laboratory Animals. The study protocol was approved by the Animal Ethics Committee at Shengjing Hospital Affiliated to China Medical University. The IACUC committee members at Institute of Shengjing Hospital Affiliated to China Medical University approved this study. All efforts were made to minimize the number of animals used and their suffering. The animals were anesthetized using an inhalant anesthetic and sacrificed by decapitation.

### Drug treatments

In both HEI-OC1 cell and isolated cochlear explant *in vitro* culture, gentamicin and NaHS were both supplemented in respective culture medium in assay specific concentrations, followed by incubation of assay specific time periods.

### MTT assay

For the measurement of metabolic activity, 3-(4,5-dimethyl-2-thiazoyl)-2, 5-diphenyltetrazolium bromide (MTT) was used according to manufacturer’s instructions (Roche). HEI-OC1 cells were seeded in 96-well plates at a density of 1000–1500 cells/well and treated with gentamicin or NaHS at dose indicated in respective figures for 48 hours, with fresh medium containing relevant drugs changed every 24 hours. 0.5 mg/ml MTT was then added to each well and incubated for 4 hours, followed by incubation with lysis buffer overnight. The optical density of solubilized formazan was measured at 570 nm on a plate reader (BioRad).

### Detection of ROS

Cellular ROS level was measured by DCFH-DA (Life Technologies) according to manufacturer’s instructions. Briefly, HEI-OC1 cells were seeded on glass coverslips placed in 6-well plates at a density of 1×10^5^ cells/well and treated with gentamicin or NaHS at dose indicated in respective figures for 48 hours, with fresh medium containing relevant drugs changed every 24 hours. Cell were then washed for 5 min twice with pre-warmed serum-free DMEM, and incubated with 5 μM DCFH-DA in serum-free medium for 30 min, followed by brief washing with PBS. Fluorescent images were taken with a fluorescent microscope. Fluorescent signal intensity was quantified from 9 randomly picked regions of interest from three independent experiments.

### Mitochondrial transmembrane potential measurement

Mitochondrial transmembrane potential (Δψm) is measured as described previously [23]. Briefly, HEI-OC1 cells were seeded in 6-well plates at a density of 1×10^5^ cells/well and treated with gentamicin or NaHS at dose indicated in respective figures for 48 hours, with fresh medium containing relevant drugs changed every 24 hours. Cells were then washed for 5 min twice with pre-warmed serum-free DMEM, and incubated with 2.5 μg/ml JC-1 (Life Technologies) for 30 min, followed by brief washing with PBS. Fluorescent signal was analyzed on a flow cytometer with 530 nm and 590 nm band pass emission filters. Mitochondrial depolarization was indicated by the ratio of the green/red fluorescence (λ530/λ590).

### Apoptosis Assay

Apoptosis was examined by co-staining with Hoechst33342 and PI (Life Technologies). HEI-OC1 cells were seeded on glass coverslips placed in 6-well plates at a density of 1×10^5^ cells/well and treated with gentamicin or NaHS at dose indicated in respective figures for 48 hours, with fresh medium containing relevant drugs changed every 24 hours. Cell were then incubated in growing medium containing 1 μg/mL Hoechst33342 and 1 μg/mL PI for 30 min, followed by brief washing with PBS. Fluorescent images were taken with a fluorescent microscope. Fluorescent signal intensity was quantified from 9 randomly picked regions of interest from three independent experiments.

### Quantitative RT-PCR

HEI-OC1 cells and/or cochlear explants were treated with gentamicin or NaHS at dose and time indicated in respective figures. Total RNA was isolated from HEI-OC1 cells and/or cochlear explants using the RNeasy MiniPrep Kit (Qiagen) according to manufacturer’s instructions. Quality and quantity of RNA and cDNA were measured by NanoDrop 8000 spectrophotometer (Thermo Scientific). 1 μg of total RNA was reverse-transcribed with Superscript II First-Strand Synthesis kit (Life Technologies) as recommended by the manufacturer. 1 μg of cDNA was amplified in 20 μL PCR reaction mixture containing the following primer pairs: Bax, forward 5’- GTT TCA TCC AGG ATC GAG CAG-3’ and reverse 5’-CAT CTT CTT CCA GAT GGT GA-3’; Bcl-2, forward 5’-CCT GTG GAT GAC TGA GTA CC-3’ and reverse 5’-GAG ACA GCC AGG AGA AAT CA-3’; Caspase-3, forward 5’-TGT CAT CTC GCT CTG GTA CG-3’ and reverse 5’-AAA TGA CCC CTT CAT CAC CA-3; GAPDH, forward 5’-AAC TTT GGC ATT GTG GAA GG-3’ and reverse 5’-GGA GAC AAC CTG GTC CTC AG-3’. GAPDH mRNA levels were measured for normalization and all data were presented as relative expression. The relative expression was analyzed using the comparative *C*t method.

### Western blot

HEI-OC1 cells or cochlear explants were treated with gentamicin or NaHS at dose and time indicated in respective figures. The cells or explants were then washed briefly with PBS and lysed in RIPA buffer (50 mM Tris, 150 mM NaCl, 0.1% SDS, 0.5% sodium deoxycholate, 1% NP-40, pH 8.0) containing protease inhibitors. The protein concentrations were detected using the BCA protein assay. Cell lysate was added into 20 μl 2x sample loading buffer (0.125 M of 5 M Tris-HCl, Amresco; 20% glycerol, Usb; 4% of 10% sodium dodecyl sulfate, Amresco; 1% β-mercaptoethanol, Amresco; 0.2% of 0.05% (w/v) bromophenol blue, Sigma) and boiled for 5 min before loading. Proteins were separated by SDS-PAGE, transferred to immobilon P membrane (Millipore), and were incubated at room temperature for 1 hour with primary antibodies against Bax, Bcl-2, Caspase-3 or GAPDH as indicated in respective figures. After incubation with primary antibodies, the membranes were washed for 10 min with PBS containing 0.1% Tween-20 thrice, and incubated with HRP-conjugated secondary antibody at room temperature for 1 hour, followed by washing for 10 min with PBS containing 0.1% Tween-20 thrice. Signals were then visualized using ECL kit (Abcam). All antibodies were purchased from Cell Signaling.

### Cochlear tissue isolation and *in vitro* culture

According to the procedure from published reports [24, 25], the timed mated pregnant mice at E13 to E14.5 were anesthetized using an inhalant anesthetic and sacrificed by decapitation. The embryos were isolated and placed in PBS (Gibco). Then unfixed bulla from embryos were dissected and incubated in calcium-magnesium-free PBS (Invitrogen) containing dispase (1 mg/ml; Invitrogen) and collagenase (1 mg/ml; Worthington) to free the cochlear duct from the surrounding condensed mesenchyme for 10–15 min. After that, the enzyme solution was replaced with Hank’s Balanced Salt Solution (HBSS, Life Technologies) and the spiral ganglia, Reissner’s membrane, and the most basal cochlear segment should be removed to obtain a flat cochlear surface preparation. For RT-PCR experiments, both the most cochlear base and the cochlear apex were removed, only the cochlear mid-turn was used. The remaining cochlear tissue was cultured on SPI filter membranes (Spi Supplies) in DMEM-F12 (Invitrogen) with B27 supplement (Invitrogen), 2.5 ng/ml FGF2 (NIH), 5 ng/ml EGF (Sigma) and 100 U/ml Penicillin (Sigma). All cultures were maintained in a 5% CO_2_/20% O_2_-humidified incubator.

### Microscopy and cochlear hair cell counts

Procedures for microscopy and hair cell counts were adapted from previously established methods [24]. The isolated cochlear explant was rinsed with PBS, fixed with 4% paraformaldehyde (PFA) in PBS for 10 min, and analyzed by fluorescent microscopy. Hair cells were identified and quantified by Atoh1/GFP expression. ImageJ software (NIH) was used to measure the length of cochlear segments, and hair cell density was calculated for each segment. Explant was also post-fixed in 1% osmium tetroxide for 2 hour and placed in 2% tannic acid twice for 30 min. The cochlea were dehydrated in a series of graded ethanol solutions and dried in a critical point drier. The specimens were fixed on a metal stage, gold-coated in a sputter coater and observed under a scanning electron microscope.

### Hair cell mechanotransduction measurement

The hair cell mechanotransduction measurements were adapted from established methods [26, 27]. Briefly, cochlear explants were treated separately with PBS, 80 μM gentamicin and 80 μM gentamicin + 250 μM NaHS for 24 hours, cochleae were excised, mounted on glass coverslips, and viewed on an Axioskop FS upright microscope (Zeiss) equipped with a 63× water-immersion objective and differential interference contrast optics. Electrophysiological recordings were performed at room temperature in solutions containing (in mM): 137 NaCl, 5.8 KCl, 10 HEPES, 0.7 NaH_2_PO_4_, 1.3 CaCl_2_, 0.9 MgCl_2_, and 5.6 D-glucose, vitamins (1:100) and amino acids (1:50) as in MEM (Invitrogen), pH 7.4 (311 mOsm/kg). Recording electrodes (2–4 MΩ) were pulled from R-6 glass (King Precision Glass) and filled with (in mM): 135 KCl, 5 EGTA-KOH, 5 HEPES, 2.5 Na_2_ATP, 2.5 MgCl_2_, and 0.1 CaCl_2_, pH 7.4 (284 mOsm/kg). The whole-cell, tight-seal technique was used to record mechanotransduction currents using a Multiclamp 700A amplifier (Molecular Devices) for cochleae. Cells were held at 75 mV, a physiologically relevant holding potential. Currents were filtered at 2–5 kHz with a low-pass Bessel filter, digitized with at least 20 kHz with a 12-bit acquisition board (Digidata 1322A or 1440A), and recorded using pClamp 8.2 software (Molecular Devices). Inner hair bundles were deflected using a stiff glass probe mounted on a PICMA chip piezo actuator (Physik Instruments) driven by a 400-mA ENV400 amplifier (Piezosystem Jena) filtered with an 8-pole Bessel filter at 10–40 kHz to eliminate residual pipette resonance.

### Statistical analysis

All data were presented as mean ± SEM from three independent experiments. Two-tailed student’s t-test was used to establish significant differences between groups. Data were determined to be statistically different when P < 0.05.

## Results

### NaHS protects viability of HEI-OC1 cells upon gentamicin exposure

**Fig 1:**
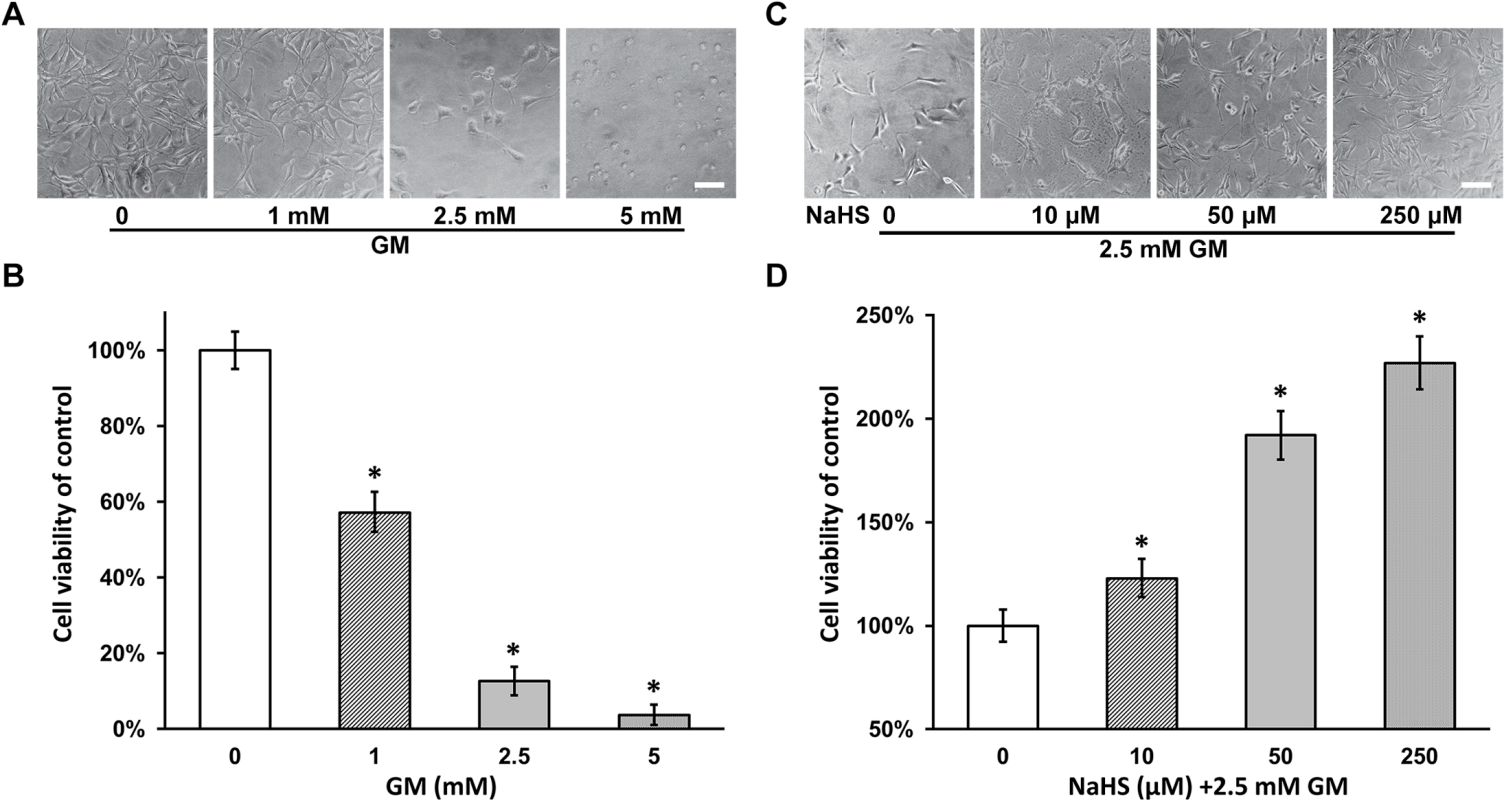
NaHS protects viability of HEI-OC1 cells upon gentamicin toxicity. (A and B) HEI-OC1 cells were cultured as described in materials and methods. Gentamicin (GM) was added to the culture medium to a concentration of 0 (control), 1, 2.5 and 5 mM respectively incubated for 48 hours. Representative culture images (A) and viability measured by MTT assay (B) of HEI-OC1 cell culture after gentamicin (GM) treatment were shown. (C and D) HEI-OC1 cells in the presence of 2.5 mM gentamicin were co-treated with 0, 10, 50 and 250 μM of NaHS respectively for 48 hours. Representative culture images (C) and viability (B) were shown. Scale bar 100 μm. Values were represented as the mean ± SEM from three independent experiments. * *P* < 0.05 vs control.

In order to establish an *in vitro* gentamicin ototoxicity model, we first exposed the HEI-OC1 cells to increasing concentrations of gentamicin in the culture medium (0, 1, 2.5 and 5 mM). As shown in Fig 1A, gentamicin treatment markedly reduced the number of cells in the culture in a dose-dependent manner. Cell viability was also significantly reduced by gentamicin exposure, as analyzed by MTT assay (Fig 1B), in a similar dose-dependent fashion. This result suggested that the use of gentamicin was able to exert toxicity to HEI-OC1 cells in our culturing conditions. Next, in order to test whether NaHS was able to protect HEI-OC1 cells from this gentamicin-induced toxicity, cells in the presence of 2.5 mM gentamicin were co-treated with 0, 10, 50 and 250 μM of NaHS respectively. We observed an obvious increase in cell numbers of NaHS-treated cultures compared to culture treated with gentamicin alone (Fig 1C). Using MTT assay, we confirmed that cell viability in the presence of NaHS was indeed significantly higher than in the absence of NaHS, in a dose-dependent manner to NaHS concentration (Fig 1D). Of note, NaHS alone (0 to 250 μM) does not affect the viability of HEI-OC1 cells in these experiments (S1 Fig). Therefore this result clearly indicated that NaHS was able to protect viability of HEI-OC1 cells upon gentamicin toxicity.

### NaHS inhibits gentamicin-induced ROS production, mitochondrial depolarization and apoptosis

**Fig 2:**
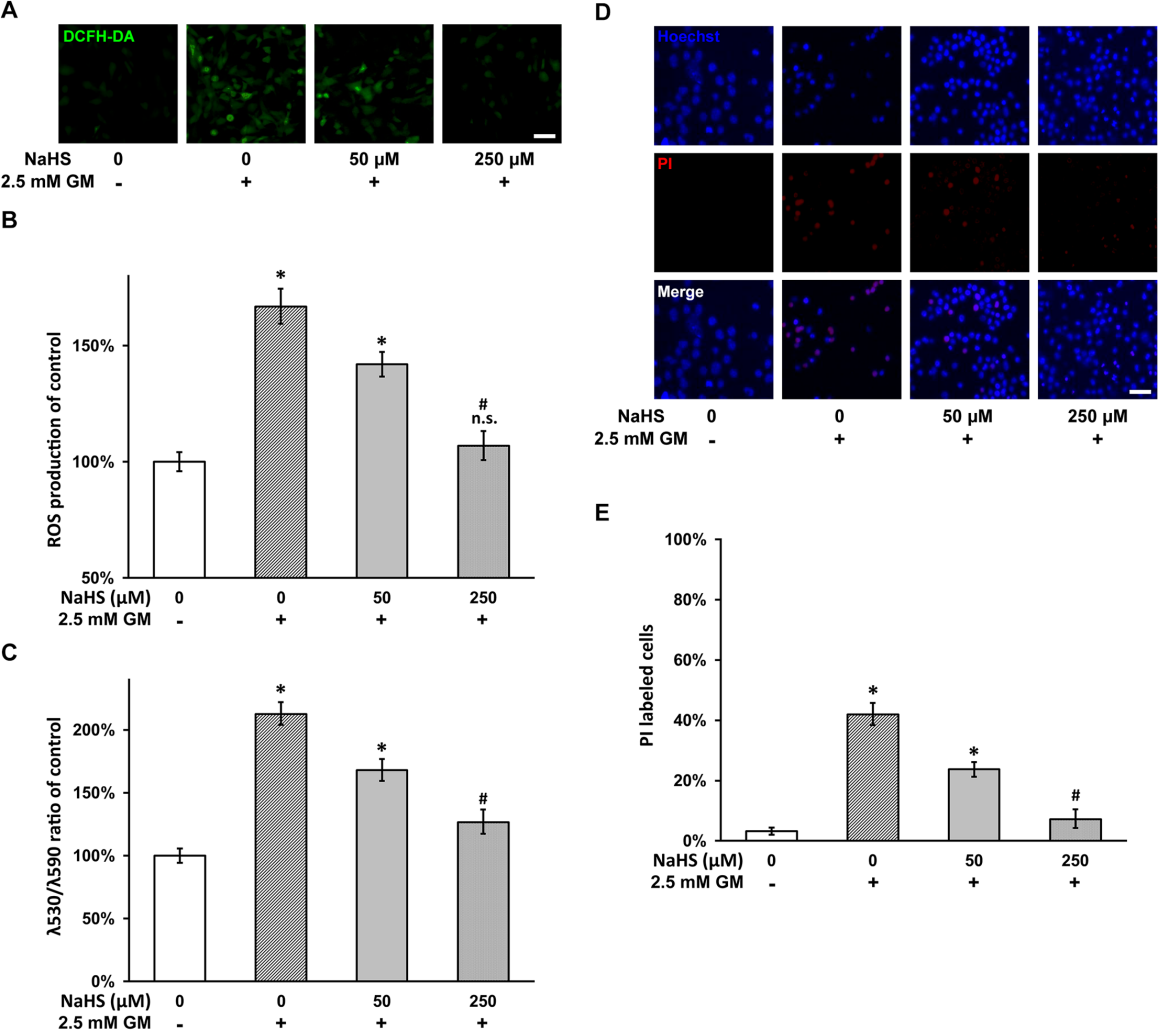
NaHS inhibits gentamicin-induced ROS production, mitochondrial depolarization and apoptosis. (A and B) HEI-OC1 cells in the presence of 2.5 mM gentamicin were co-treated with 0, 50 and 250 μM of NaHS respectively for 48 hours. Representative DCFH-DA (green) staining fluorescence images (A) and quantification of fluorescent intensity (B) were shown. (C) Mitochondrial depolarization was measured in HEI-OC1 cells as treated in (A). (D and E) Hoechst (blue)/PI (red) staining (D) and percentage of PI-positive apoptotic cells (E) in HEI-OC1 culture treated as in (A) were shown. Scale bar 100 μm. Values were represented as the mean ± SEM from three independent experiments. * *P* < 0.05 vs control. n.s. not significant vs control. # P < 0.05 vs 0 and 50 μM NaHS treatment.

As gentamicin exposure was reported to result in the production of ROS in *in vitro* hair cell cultures [28], we next investigated whether NaHS could inhibit this process in HEI-OC1 cell culture. Similarly HEI-OC1 cells in the presence of 2.5 mM gentamicin were co-treated with 0, 50 and 250 μM of NaHS, and DCFH-DA fluorescence was performed to determine ROS production (see Materials and Methods). As expected, gentamicin insult caused a visible elevation of DCFH-DA fluorescence in HEI-OC1 cell culture, compared with untreated cells (Fig 2A, left two panels). The addition of 50 μM NaHS in the gentamicin-treated culture resulted in a noticeable reduction in DCFH-DA fluorescence, and 250 μM of NaHS has brought DCFH-DA signal further down to the level of cells untreated with gentamicin (Fig 2A, right two panels). Quantification of DCFH-DA in the above cultures also indicated that NaHS was able to dose-dependently inhibit ROS production following gentamicin exposure (Fig 2B).

Gentamicin functions to inhibit protein synthesis in the mitochondrial ribosomes [29] that leads to the opening of mitochondrial permeability transition pores and in turn mitochondrial depolarization [30]. Indeed we found that in HEI-OC1 cells, gentamicin significantly increased mitochondrial depolarization, and co-treatment with increasing concentration of NaHS was able to inhibit the gentamicin-induced mitochondrial depolarization in a dose-dependent manner (Fig 2C). We further investigated if the same NaHS treatment was able to inhibit apoptosis, since mitochondrial depolarization causes release of pro-apoptotic factors from inter-membrane space. In a Hoechst/PI staining experiment, 2.5 mM gentamicin greatly increased the percentage of PI-positive cells, indicative of elevated apoptosis (Fig 2D and 2E). As expected, NaHS treatment was found to inhibit gentamicin-induced apoptosis in a dose-dependent manner, with 250 μM of NaHS almost reduced the percentage of PI-positive cells to the level without gentamicin insult (Fig 2D and 2E).

### NaHS inhibits gentamicin-induced intrinsic apoptotic pathway in HEI-OC1 cells

**Fig 3:**
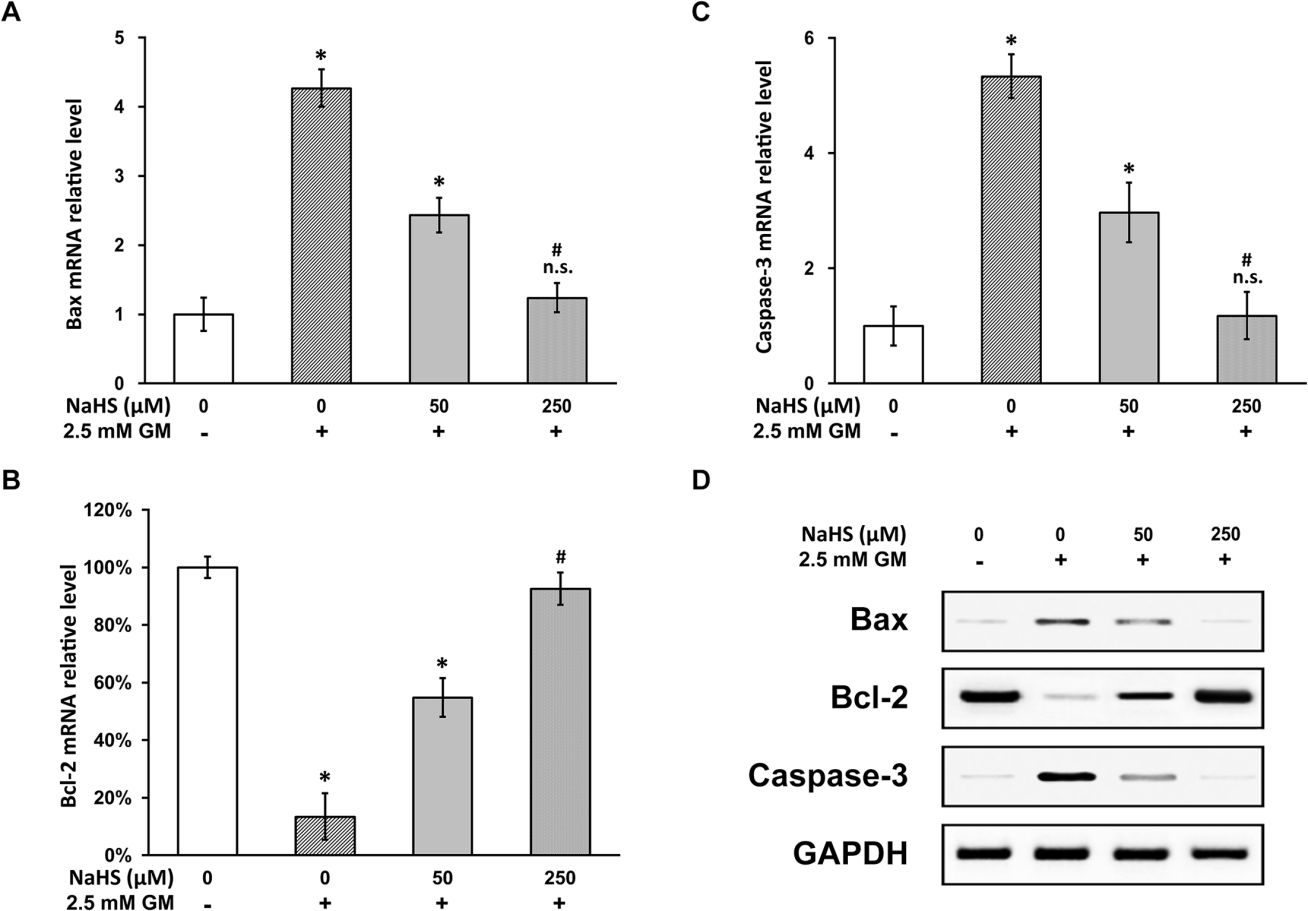
NaHS reverses expressions of gentamicin-induced intrinsic apoptotic pathway factors in HEI-OC1 cell culture. (A to C) mRNA levels of Bax (A), Bcl-2 (B) and Caspase-3 (C) were examined with RT-PCR in HEI-OC1 cells treated as in Fig 2. Values were represented as the mean ± SEM from three independent experiments, normalized to GAPDH mRNA levels. * *P* < 0.05 vs control. n.s. not significant vs control. # P < 0.05 vs 0 and 50 μM NaHS treatment. (D) Protein levels of Bax, Bcl-2 and Caspase-3 were examined with western blot analysis in HEI-OC1 cells treated as in Fig 2.

Having established the anti-apoptotic effect of NaHS in gentamicin-induced HEI-OC1 ototoxicity model, we next set to investigate the underlying molecular mechanism. The intrinsic apoptotic pathway is regulated by the pro-apoptotic factor Bax and the anti-apoptotic factor Bcl-2, and their combined action induces caspase-3 dependent apoptosis [9, 13]. Using RT-PCR, we examined the mRNA levels of the above genes, and found that gentamicin induced apoptosis through the intrinsic pathway, evident by increased Bax (Fig 3A) and decreased Bcl-2 (Fig 3B), as well as much elevated caspase-3 (Fig 3C) expressions. More importantly, NaHS indeed was able to attenuate the expression of these gentamicin-induced intrinsic apoptotic pathway factors in a dose-dependent manner (Fig 3A, 3B and 3C), which explains its anti-apoptotic effect described earlier in this study. As expected, protein expressions of the involved intrinsic apoptotic factors were found to behave exactly as their messenger levels (Fig 3D).

### NaHS protects hair cells in mouse cochlear explant from gentamicin-induced ototoxicity

**Fig 4:**
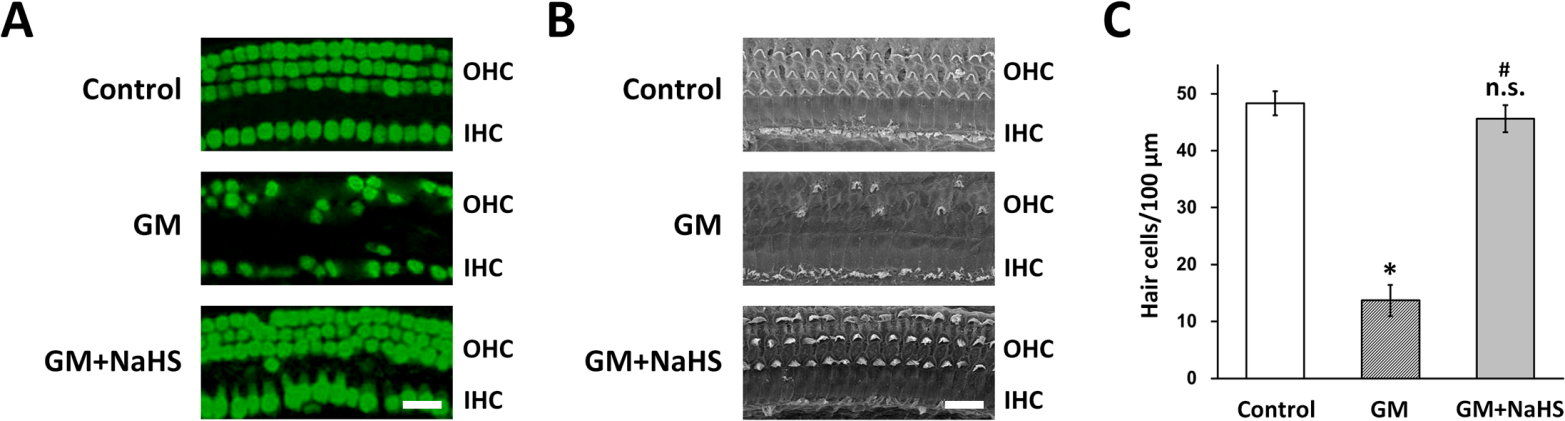
NaHS inhibits gentamicin-induced ototoxicity in mouse cochlear explant. Mouse cochlear explant was isolated and cultured as described in materials and methods. After 24 hours cultured in the incubator, cochlear explant were divided into 3 treatment groups: PBS (control), 80 μM gentamicin (GM) and 80 μM gentamicin + 250 μM NaHS (GM+NaHS) for 24 hours. (A) Hair cells from mid apex segment of cochlear showing outer hair cells (OHC) and inner hair cells (IHC) labeled with Atoh1/GFP (green). Scale bar 25 μm. (B) Scanning electron micrographs of the mid apex segment from cochlear explant as treated in (A). (C) Quantification of hair cells in three cochlear explant groups. Values were represented as the mean ± SEM from three independent experiments. * *P* < 0.05 vs control. n.s. not significant vs control. # P < 0.05 vs GM.

The above results clearly demonstrated that, in cultured HEI-OC1 cells upon gentamicin insult, NaHS was able to protect cell viability and inhibit ROS production, mitochondrial depolarization and apoptosis. More importantly, NaHS treatment inhibited gentamicin-induced intrinsic apoptotic pathway. To further validate our findings, we next performed similar experiments using isolated mouse cochlea tissues. Mouse cochlear explants were isolated and cultured as described in materials and methods. As shown in Fig 4, they were then divided into 3 treatment groups: PBS (Control), 80 μM gentamicin alone (GM) and 80 μM gentamicin + 250 μM NaHS (GM+NaHS). By both optical and scanning electron microscopy, we found that compared with PBS control, 80 μM gentamicin treatment significantly reduced the number of hair cells in the cultured cochlear explant (Fig 4A and 4B). Importantly, simultaneous treatment of gentamicin with 250 μM NaHS significantly inhibited the gentamicin-induced reduction in hair cell number in the cochlear explant, to a level comparable to the PBS control treated group (Fig 4C). This result was consistent with our earlier finding using the HEI-OC1 cells, which suggested that NaHS was likely to function very similarly in the two gentamicin insult models.

### NaHS inhibits gentamicin-induced intrinsic apoptotic pathway in cochlear explant

**Fig 5:**
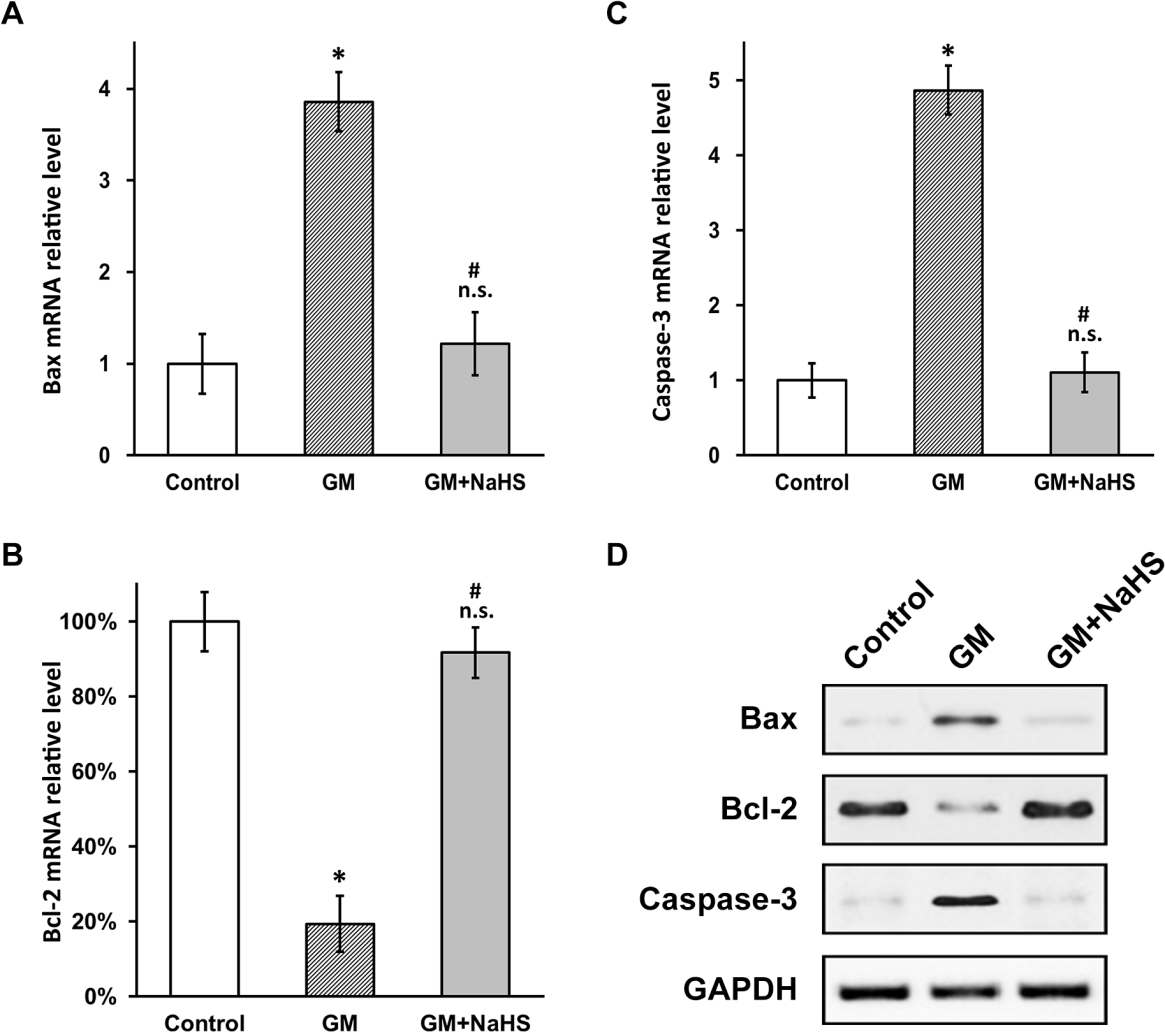
NaHS reverses gentamicin-induced apoptotic gene expressions in cochlear explant. (A to C) mRNA levels of Bax (A), Bcl-2 (B) and Caspase-3 (C) were examined with RT-PCR in cochlear explant treated as in Fig 4. Values were represented as the mean ± SEM from three independent experiments, normalized to GAPDH mRNA levels. * *P* < 0.05 vs control. n.s. not significant vs control. # P < 0.05 vs GM. (D) Protein levels of Bax, Bcl-2 and Caspase-3 were examined with western blot analysis in cochlear explant treated as in Fig 4.

Next similar as in HEI-OC1 cells, we examined the mRNA levels of intrinsic apoptotic pathway factors Bax, Bcl-2 and caspase-3 in the three experimental groups of isolated cochlea treated as in Fig 4. We found that gentamicin insult caused the exact same changes in the expressions of the above genes, with Bax and caspase-3 increased and Bcl-2 decreased significantly relative to the PBS control group (Fig 5A, 5B and 5C). As expected, in gentamicin and NaHS co-treatment group, their relative mRNA (Fig 5A, 5B and 5C) and protein (Fig 5D) levels were brought back to those of respective PBS control, strongly indicating that NaHS was also able to function as an anti-apoptotic agent in isolated cochlea tissues to protect against gentamicin ototoxicity.

### NaHS protects mechanotransduction activity in gentamicin-insulted cochlear explant

**Fig 6:**
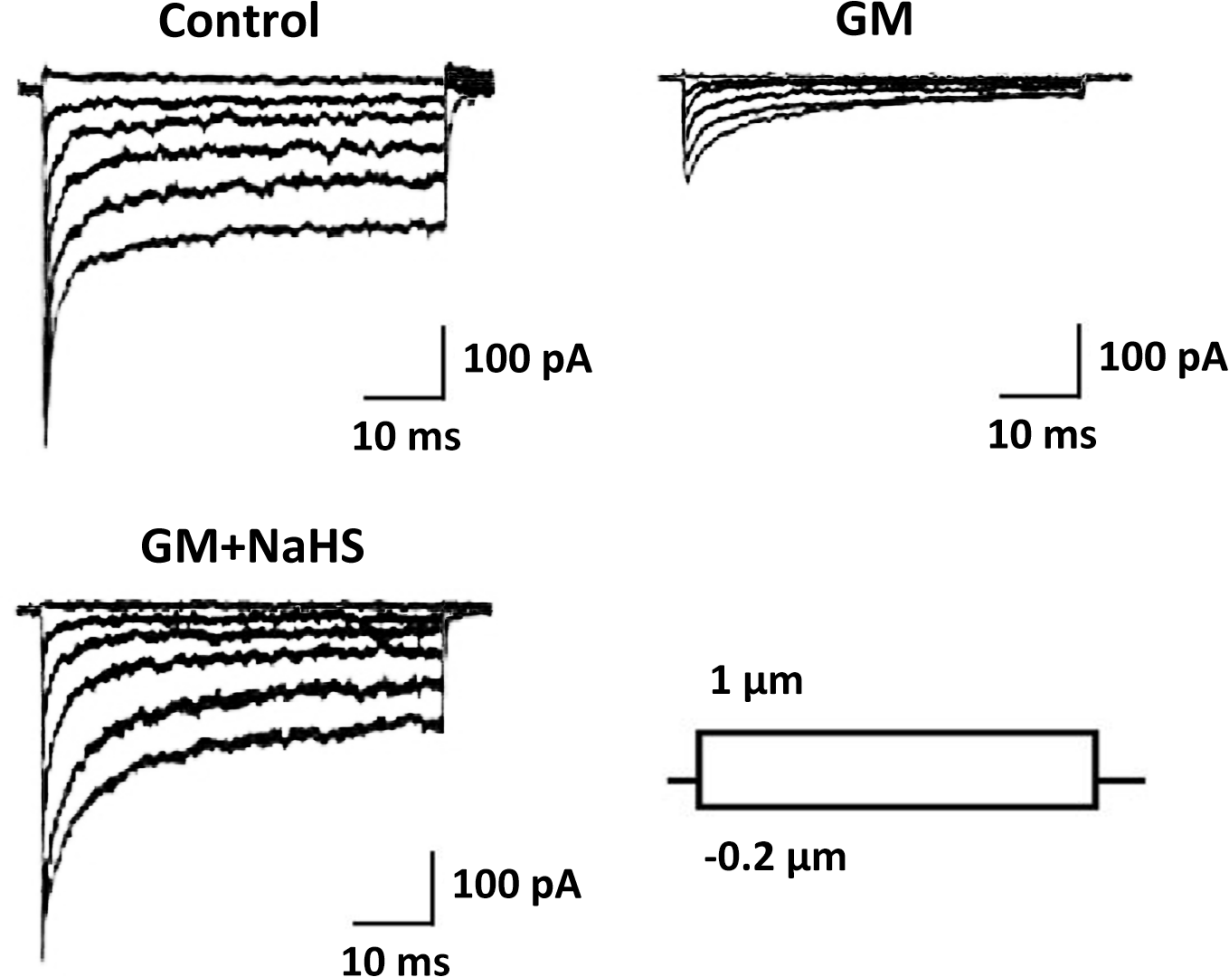
NaHS protects mechanotransduction activity of cochlear explant from gentamicin-induced ototoxicity. Mechanotransduction currents were recorded at −75 mV as described in materials and methods, from the mid apex segment of cochlear explant bathed in 1.3 mM Ca^2+^, treated as in Fig 4. Stimulus protocol and scale bar applies to all current families.

Hair cells serve as mechanotransduction amplifier in the cochlea by converting sound vibrations to neural electric impulses. Therefore we further assessed whether NaHS was able to protect cochlear explants from gentamicin, and retain their mechanosensory functions, by recording mechanotransduction currents from the cochlear hair cells in the three experimental groups. As shown in Fig 6, the cochlear tissue treated with 80 μM gentamicin had significantly attenuated transduction currents, compared to the PBS control group. However, the cochlea co-treated with both 80 μM gentamicin and 250 μM NaHS exhibited transduction currents as normal as the PBS control, further demonstrating that NaHS was able to effectively protect mechanotransduction activity of cochlea from gentamicin insult.

Taken together our results, using both HEI-OC1 cell line and *in vitro* cultured cochlea model, provided strong evidence that, apart from protecting cochlea from gentamicin-induced structural lesions, NaHS also protected the hair cells from gentamicin-induced mechanotransduction damages.

## Discussion

To summarize, first by using the gentamicin-insulted HEI-OC1 cell model, we have established that gentamicin reduced cell viability and elevated cellular ROS production, which in turn triggered mitochondrial depolarization and consequently activation of the intrinsic apoptotic pathway. More importantly, our results demonstrated that NaHS was able to protect HEI-OC1 cell from gentamicin-induced toxicity in a dose-dependent manner. Moreover by using isolated mouse cochlear culture, we validated the anti-apoptotic effect of NaHS, evident by reversed expression profiles of intrinsic apoptotic factors as well as protected hair cell mechanotransduction activity.

Gentamicin was found to induce oxidative stress in hair cells that could eventually lead to apoptosis [7, 28, 31], which severely limited its clinical use. Aminoglycosides such as gentamicin tend to accumulate in the mitochondria of the hair cells and cause increased production of ROS [32], which are found to be the major contributor of aminoglycoside-induced hearing loss [16]. The accumulation of ROS in the mitochondria of hair cells reduces its antioxidant defense, triggering mitochondrial depolarization and release of cytochrome *c* which activates caspase-3 and initiates apoptosis [33]. NaHS most likely exerts its protective functions against gentamicin by alleviating cellular oxidative stress, which in turn attenuates apoptosis. Our current study indicated that NaHS significantly reduced cellular ROS production in gentamicin-treated HEI-OC1 cell culture. This observation is consistent with NaHS being an oxygen scavenger. We speculate that, since accumulation of ROS triggers apoptotic cell death in hair cells, NaHS also functions as a cellular ROS scavenger and significantly reduces ROS production following gentamicin ototoxicity, to protect hair cells. Indeed our results clearly demonstrated that, by reducing ROS levels, NaHS also significantly inhibited gentamicin-induced mitochondrial depolarization in HEI-OC1 cells, as well as greatly attenuated apoptosis in both HEI-OC1 cell and isolated mouse cochlear cultures.

We also investigated the apoptotic pathway accounting for gentamicin-induced hair cell loss, and found that the intrinsic pathway factors Bax and Bcl-2 were affected by gentamicin treatment. The pro-apoptotic factor Bax and the anti-apoptotic factor Bcl-2 both belong to the Bcl-2 family proteins, and their combined action were found to trigger the opening of inner mitochondrial permeability transition pore [9, 13]. Opening of mitochondrial permeability transition pore also leads to mitochondrial depolarization and release of cytochrome *c* [12], and in turn activates the caspase-3 dependent apoptotic pathway [13]. Interestingly, NaHS was also found to inhibit the gentamicin-induced expression profile changes of Bax, Bcl-2 and caspase-3, therefore preventing apoptosis through the intrinsic pathway in HEI-OC1 cell and isolated mouse cochlear cultures following gentamicin insult. This result of ours uncovers for the first time the molecular mechanism underlying the anti-apoptotic effect of NaHS.

In conclusion, we hereby presented that NaHS exhibited significant effect in protection against gentamicin-induced ototoxicity, through inhibiting production of ROS and caspase-3 dependent intrinsic apoptosis. By employing both HEI-OC1 cell line and isolated cochlear *in vitro* culture of gentamicin ototoxicity, our experiments were also consistently reproduced in these different models. Finally, our study provided the first instance suggesting the potential of NaHS as a therapeutic agent to protect against aminoglycoside-induced hearing loss.

## Supporting Information

S1 Fig Effect of NaHS alone on cell viability of HEI-OC1 cells. HEI-OC1 cells were treated with 0, 10, 50, 250, 500 μM of NaHS for 48 hours and subject to viability measurement by MTT assay. Values were represented as the mean ± SEM from three independent experiments. n.s. not significant vs control. * P < 0.05 vs control.
(TIF)
