# Supplementary figures and images for: NaHS Protects Cochlear Hair Cells from Gentamicin-Induced Ototoxicity by Inhibiting the Mitochondrial Apoptosis Pathway
Source: PLoS One. 2015 Aug 21;10(8):e0136051. doi: 10.1371/journal.pone.0136051 (PMC4546415; doi:10.1371/journal.pone.0136051)

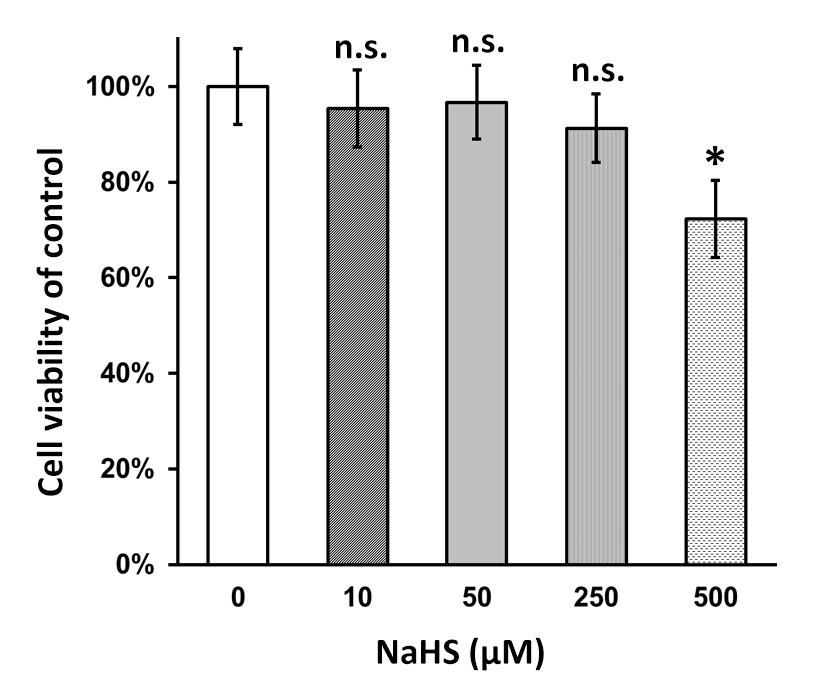

Supplement: S1 Fig — HEI-OC1 cells were treated with 0, 10, 50, 250, 500 μM of NaHS for 48 hours and subject to viability measurement by MTT assay. Values were represented as the mean ± SEM from three independent experiments. n.s. not significant vs control. * P < 0.05 vs control. (TIF) [file pone.0136051.s001.tif]
